# Toward computational modelling on immune system function

**DOI:** 10.1186/s12859-020-03897-5

**Published:** 2020-12-14

**Authors:** Francesco Pappalardo, Giulia Russo, Pedro A. Reche

**Affiliations:** 1 Department of Drug and Health Sciences, University of Catania, V.le A. Doria 6, 95125 Catania, Italy; 2 Departamento de Immunología (Microbiología I), Universidad Complutense de Madrid, Facultad de Medicina, Plaza Ramón y Cajal, 28040 Madrid, Spain

## Abstract

The 3rd edition of the computational methods for the immune system function workshop has been held in San Diego, CA, in conjunction with the IEEE International Conference on Bioinformatics and Biomedicine (BIBM 2019) from November 18 to 21, 2019. The workshop has continued its growing tendency, with a total of 18 accepted papers that have been presented in a full day workshop. Among these, the best 10 papers have been selected and extended for presentation in this special issue. The covered topics range from computer-aided identification of T cell epitopes to the prediction of heart rate variability to prevent brain injuries, from In Silico modeling of Tuberculosis and generation of digital patients to machine learning applied to predict type-2 diabetes risk.

## Introduction

In recent years, a growing openness by pharmaceutical regulatory agencies to receive and accept evidence obtained in silico, i.e. through the use of computational modelling and simulation platforms, has further increased interest in the field of computational immunology and its derived instruments and methodologies. Nowadays, the possibility of replacing in vitro and in vivo experiments with computer simulations is no longer a chimera. Both data-driven and mechanistic models can be used to suggest novel biomarkers, to predict the occurrence of heart attacks, to select best epitopes for novel vaccines, to test novel and existing pharmaceutical compounds by means of computer simulations, to realize “virtual patients” that can be enrolled into clinical trials in addition to real patients for augmenting statistical evidence.

Consequently, the idea of a workshop on these topics remains one of our major priorities, as it is starting to represent a regular point for discussion and comparison among researchers coming from all over the world. From the 18 accepted workshop contributions, 10 papers have been extended for inclusion in this special issue. Topics of interest cover different fields, from machine learning methods for type-2 diabetes and selection of T cell epitopes, to the simulation of diseases such as yellow-fever and Tuberculosis.

We believe that the application of computer methods to immunology will become increasingly important, and this is especially true in the era of the COVID-19 pandemic, which has shown how timely measures to design, test and accelerate the development of treatments may save many human lives. To this end, we expect consistent participation even for the 4th edition of the workshop that will be held entirely online, from December 16th to 19th, 2020, always under the umbrella of the IEEE International Conference on Bioinformatics and Biomedicine (BIBM 2020).

## Topics covered

The main topics of interest included the simulation of pathologies involving the immune system as a solution or cause of the disease, as well as contributions about the optimization of simulation methodologies, about the definition of novel vaccine candidates using computational methods, methods based on statistical techniques, machine learning, synthetic data and wearable health devices, meta-heuristics. Moreover, the topic of in silico trials is strongly highlighted: in silico trials innovations represent a powerful pipeline for the prediction of the effects of specific therapeutic strategies and related clinical outcomes.

SARS-CoV-2 is a severe respiratory infection that infects humans. Its outburst entitled it as a pandemic emergence. To get a grip on this outbreak, specific preventive and therapeutic interventions are urgently needed. In their work, Russo et al. [1], present an in silico platform that showed to be in very strong agreement with the latest literature in predicting SARS-CoV-2 dynamics and related immune system host response. The computational framework they described, namely Universal Immune System Simulator (UISS), is potentially ready to be used as an in silico trial platform to predict the outcome of vaccination strategy against SARS-CoV-2.

In 2018, about 10 million people were found infected by tuberculosis, with approximately 1.2 million deaths worldwide. Although these numbers have been relatively stable in recent years, tuberculosis is still considered one of the top 10 deadliest diseases worldwide. In this context, the EU and Indian DBT funded project STriTuVaD—In Silico Trial for Tuberculosis Vaccine Development—is supporting the identification of new interventional strategies against tuberculosis thanks to the use of Universal Immune System Simulator (UISS), a computational framework capable of predicting the immunity induced by specific drugs such as therapeutic vaccines and antibiotics. In their research [2] authors present how their in silico trials platform accurately simulates tuberculosis dynamics and its interaction within the immune system, and how it predicts the efficacy of the combined action of isoniazid and RUTI vaccine in a specific digital population cohort.

Stolfi et al. [3] investigate the mechanisms involved in the onset of type 2 diabetes in the absence of familiarity. This has led to the development of a computational model that recapitulates the aetiology of the disease and simulates the immunological and metabolic alterations linked to type-2 diabetes subject to clinical, physiological, and behavioural features of prototypical human individuals. The paper analysed the time course of 46,170 virtual subjects experiencing different lifestyle conditions. The resulting machine learning model adequately predicts the synthetic dataset and can, therefore, be used as a computationally-cheaper version of the detailed mathematical model, ready to be implemented on mobile devices to allow self-assessment by informed and aware individuals.

In their research, Gomez-Perosanz et al. [4], assembled an epitope dataset consisting of 844 unique virus-specific CD8+ T cell epitopes and their source proteins. Then, they analyzed cleavage predictions by PCPS immunoproteasome cleavage model on this dataset and compared them with those provided by a related method implemented by NetChop web server. Following these results, authors tuned the PCPS web server to predict CD8+ T cell epitopes and predicted the entire SARS-CoV-2 epitope space. An improved version of PCPS named iPCPS for predicting proteasome cleavage sites and peptides with CD8+ T cell epitope features was the final goal the authors presented.

Prediction of patient outcome in medical intensive care units (ICU) may help for development and investigation of early interventional strategies. Several ICU scoring systems have been developed and are used to predict clinical outcome of ICU patients. These scores are calculated from clinical physiological and biochemical characteristics of patients. Heart rate variability (HRV) is a correlate of cardiac autonomic regulation and has been evident as a marker of poor clinical prognosis. In their work, Zhang et al. [5] found a feature extraction strategy that was applied to measure the HRV fluctuation during time. A prediction model was developed based on HRV measures with genetic algorithm (GA) for feature selection. The result was compared with earlier reported scoring systems, encouraging further development and practical application. These findings may help predict the brain injury patient outcome more effectively than the previously adopted illness severity scores.

An effective yellow fever (YF) vaccine has been available since 1937. Nevertheless, questions regarding its use remain poorly understood, such as the ideal dose to confer immunity against the disease, the need for a booster dose, the optimal immunisation schedule for immunocompetent, immunosuppressed, and pediatric populations, among other issues. In their work, Bonin et al. [6] aim to demonstrate that computational tools can be used to simulate different scenarios regarding YF vaccination and the immune response of individuals to this vaccine, thus assisting the response of some of these open questions.

The immune checkpoint receptor programmed cell death protein I (PD-1) has been identified as a key target in immunotherapy. PD-1 reduces the risk of autoimmunity by inducing apoptosis in antigen-specific T cells upon interaction with programmed cell death protein ligand I (PD-L1). Various cancer types overexpress PD-L1 to evade the immune system by inducing apoptosis in tumor-specific CD8+ T cells. Roither et al. [7] present the results of the first molecular dynamics simulations of PD-1 with a complete extracellular domain, focusing on the role of the BC-loop of PD-1 upon binding PD-L1 or nivolumab. Due to the structural differences of the BC-loop, a signal transduction based on active conformation cannot be ruled out. These findings will have an impact on drug design and will help to refine immunotherapy blocking antibodies.

Multiple sclerosis (MS) represents nowadays in Europe the leading cause of non-traumatic disabilities in young adults, with more than 700,000 EU cases. Although huge strides have been made over the years, MS etiology remains partially unknown. Furthermore, the presence of various endogenous and exogenous factors can greatly influence the immune response of different individuals, making it difficult to study and understand the disease. Pernice et al. [8] propose a new model to study the immune response in relapsing remitting MS (RRMS), the most common form of MS that is characterized by alternate episodes of symptom exacerbation (relapses) with periods of disease stability (remission). In this new model, both the peripheral lymphonode/blood vessel and the central nervous system are explicitly represented. The model was created and analysed using Epimod, our recently developed general framework for modeling complex biological systems.

In their work, Zhang et al. have developed an important data warehouse, TANTIGEN 2.0, that collects accessible website information from a large number of studies. Integrated computational analysis tools in TANTIGEN 2.0 enable users to combine data and domain knowledge, use tailored bioinformatics tools, and simulate experiments. It represents a rich information resource for the study of cancer immunology and immunotherapy.

One of the most challenging tasks for using virtual patients is developing a methodology to reproduce biological diversity of the target population, ie, providing an appropriate strategy for generating libraries of digital patients. This has been achieved through the creation of the initial immune system repertoire in a stochastic way, and through the identification of a vector of features that combines both biological and pathophysiological parameters, personalising the digital patient to reproduce the physiology and the pathophysiology of the subject. In their work, Juarez et al. [9], propose a sequential approach to sampling from the joint features population distribution for the creation of a cohort of virtual patients with some specific characteristics. This resembles the recruitment process for the target clinical trial, which then can be used for augmenting the information from the physical the trial to help reduce its size and duration.

## Conclusion

We are encouraged by the growing support that the CMISF workshop is obtaining, and we believe that such support will become increasingly stronger, as the use of of computational methods and approaches to be used in the future in Silico Trials will become a common practice in the developing of new drugs. EMA and FDA have in fact expressed interest in the use of computational tools for the qualification of new drugs, and they are now moving towards the definition of the standards and rules to include computational models inside the qualification pipeline. To ensure that such themes are covered, we also included novel methods and approaches for in Silico Trials as a prominent topic of interest of the upcoming 4th CMISF workshop that will be held online due to COVID-19 issues.
